# Psychological Predictors of Symptom Severity in Disorders of Gut–Brain Interaction: A Machine Learning Approach

**DOI:** 10.1192/j.eurpsy.2026.10645

**Published:** 2026-07-31

**Authors:** S. Yoon, W.-M. Bahk, S.-Y. Lee, B.-H. Yoon, E.-S. Lim

**Affiliations:** 1 Department of Psychiatry, Wonkwang University Hospital, Iksan; 2 Department of Psychiatry, The Catholic University of Korea, Seoul; 3 Department of Psychiatry, Naju National Hospita, Naju; 4 Department of Psychiatry, Shinsegae Hyo Hospital, Gimje, Korea, Republic Of

## Abstract

**Introduction:**

Disorders of gut–brain interaction (DGBI), including irritable bowel syndrome (IBS), functional dyspepsia (FD), and functional constipation (FC), are prevalent conditions associated with psychological comorbidities. While psychosocial correlates of symptom burden have been widely reported, machine learning approaches to phenotype severity and predict risk based on psychological profiles remain limited.

**Objectives:**

This study aimed (1) to identify severity-based clinical phenotypes of DGBI patients using unsupervised clustering of gastrointestinal symptom indices, and (2) to evaluate the predictive value of psychological variables in differentiating severity groups using machine learning.

**Methods:**

A total of 373 patients diagnosed with DGBI at a Brain-gut axis Clinic, Department of Psychiatry, Wonkwang university hospital from 2020 to 2023 completed validated gastrointestinal severity scales (IBS-SSS, NDI-K, PAC-SYM, GERD-Q) and psychological assessments (BDI, BAI, CTQ, MSPSS, CD-RISC, DS-14). K-means clustering (k=3) was applied to GI severity measures to define mild, moderate, and severe groups. Group comparisons of psychological measures were conducted using ANOVA/Welch’s ANOVA with Benjamini–Hochberg correction. Random Forest classifiers with nested 5-fold cross-validation and SMOTE oversampling were used to predict severity clusters. Feature importance was assessed by permutation analysis.

**Results:**

Clustering classified patients into mild (n=159), moderate (n=132), and severe (n=82) groups. Significant between-group differences were observed in CTQ subscales (physical, sexual, emotional abuse; all FDR-corrected *p* < 0.01) and BAI muscular/motoric symptoms (*p* = 0.0066). Machine learning models based on CTQ or BAI alone showed modest performance (accuracy ~52–57%). Combining CTQ+BAI improved accuracy to 60.6% (F1=58.4%). The model incorporating all psychological variables achieved the best performance (accuracy 65.4%, F1=64.1%). Permutation importance highlighted sexual and emotional abuse as top predictors.

**Conclusions:**

Our findings indicate that early adverse experiences and anxiety-related symptoms strongly differentiate severity phenotypes in DGBI. Machine learning models integrating multiple psychological dimensions outperform single-scale approaches, supporting precision psychosomatic profiling for DGBI management.

**Disclosure of Interest:**

None Declared

